# Baduanjin Exercise for Type 2 Diabetes Mellitus: A Systematic Review and Meta-Analysis of Randomized Controlled Trials

**DOI:** 10.1155/2017/8378219

**Published:** 2017-10-19

**Authors:** Junmao Wen, Tong Lin, Yinhe Cai, Qianying Chen, Yuexuan Chen, Yueyi Ren, Senhui Weng, Boqing Wang, Shuliang Ji, Wei Wu

**Affiliations:** ^1^Guangzhou University of Chinese Medicine, Guangzhou, China; ^2^Department of Cardiovascular Disease, First Affiliated Hospital of Guangzhou University of Chinese Medicine, Guangzhou, China

## Abstract

**Objective:**

To investigate the effects of Baduanjin exercise for type 2 diabetes mellitus.

**Methods:**

Literature retrieval was performed in several databases, including PubMed, EMBASE, Cochrane Library, CNKI, Wanfang Data Information Site, CBM, and VIP from inception to April 2017. Randomized controlled trials on evaluating the effects of Baduanjin exercise were identified. The primary outcomes were glycosylated hemoglobin, fasting blood-glucose, and postprandial plasma glucose. Review Manager 5.2 (RevMan 5.2) and Stata V.13.0 software were conducted for data analysis.

**Results:**

The results of the meta-analysis indicated that the effects of type 2 diabetes mellitus were favoring Baduanjin plus conventional therapy, when compared with the routine treatment. Baduanjin plus conventional therapy lowered the level of glycosylated hemoglobin, fasting blood-glucose, postprandial plasma glucose, TC, TG, and LDL-C and improved HDL-C. Adverse events were not mentioned in all included studies. No publication bias was detected by Begg's and Egger's test and no single study affected the overall result by influence analysis.

**Conclusions:**

Evidence from meta-analysis suggested that Baduanjin exercise plus conventional therapy has a positive effect on type 2 diabetes mellitus. However, more rigorously designed and large sample RCTs are required to confirm the efficacy and safety in further studies.

## 1. Introduction

### 1.1. Description of the Condition

Caused by multiple aetiology, diabetes mellitus is a metabolic disorder which is characterized by chronic hyperglycemia resulting from the deficiency of insulin secretion and/or insulin action [1]. The amount of people with diabetes mellitus across countries and regions in 2015 and 2040 (20–79 years) was estimated at 415 million and 642 million, respectively [2]. Among the three main types of diabetes mellitus, type 2 diabetes mellitus (T2DM) is the most prevalent form and has increased with the development of society [2]. Approximately 87% to 91% of people with diabetes mellitus are estimated to have T2DM in high-income countries [3–6]. The diabetic is at high risk for experiencing complications such as cardiovascular disease, kidney disease, and diabetic neuropathy [7]. The international economy is suffering a great loss due to the increasing of the complications of diabetes mellitus, such as morbidity, disability, and mortality, especially in the developing countries [8]. As a nondrug and cost-effective intervention of T2DM, physical activities have received attention. It is well established that physical activities including aerobic exercise and resistance exercise [9] can decrease long-term morbidity and mortality and increase the body's sensitivity to insulin [10–12].

### 1.2. Description of the Intervention

Modern pharmacological researches proved that traditional Chinese medicine (TCM) may be effective for T2DM through lowering blood-glucose level in multichannel and multitargeting way [13, 14]. As an inherent component of TCM, Baduanjin (the Eight Section Brocades) is a traditional cultivation health method which can be easy to administrate [15] and a practical intervention which can exert positive effects on the conditions of fatigue-predominant subhealth (FPSH) [16], Parkinson's disease [17], hypertension [18], knee osteoarthritis (OA) [19], and dyslipidemia [20]. Moreover, increasing evidence from randomized controlled trials (RCTs) shows that Baduanjin is promising as an alternative intervention or therapy for T2DM [21]. However, critical evidence which confirms the clinical value of Baduanjin in patients with T2DM is still insufficient. Therefore, we conduct a meta-analysis of available literature to evaluate the efficacy of Baduanjin for the treatment of T2DM.

## 2. Methods

### 2.1. Eligibility Criteria

*Types of Studies*. Our study included randomized controlled trials (RCTs) for evaluating the efficacy of Baduanjin exercise in type 2 diabetes mellitus regardless of the length of treatment.

*Types of Participants*. Participants of any age and gender who are diagnosed with diabetes based on Diagnosis and Classification of Diabetes Mellitus Provisional Report of a WHO Consultation are included [16].

*Types of Interventions*. Patients of control group were given conventional therapy including health education, routing nursing, and oral antidiabetic drugs. In addition to conventional therapy, the patients of the treatment group were taking Baduanjin exercise regardless of duration.

*Types of Outcome Measure*. The primary outcome measures were glycosylated hemoglobin, fasting blood-glucose, and postprandial plasma glucose. The secondary outcome measures were (1) blood lipids: total cholesterol (TC), triglyceride (TG), high-density lipoprotein cholesterol (HDL-C), and low-density lipoprotein cholesterol (LDL-C); (2) adverse events.

### 2.2. Literature Search

We performed literature retrieval electronically in the following databases: PubMed, EMBASE, Cochrane Library, Chinese National Knowledge Infrastructure (CNKI), Wanfang Data Information Site, Chinese Biomedical Database (CBM), and Chinese Science and Technique Journals Database (VIP). All of the searches ended before April 2017. The search terms are as follows: (“Baduanjin” OR “Baduanjin exercise” OR “baduanjin” OR “eight section brocades” OR “Qigong”) AND (“type 2 diabetes” OR “Non-insulin-dependent diabetes mellitus”). These terms were translated into Chinese when retrieving Chinese database.  Table 1 showed the search strategy for PubMed.

### 2.3. Study Selection and Data Extraction

The included studies were randomized clinical trials (RCTs). Articles were excluded if incomplete data on outcome measures could be extracted, information were inadequate to require, or intervention included any other traditional Chinese medicine (TCM) therapy. Moreover, we excluded animal experiments, expert experience, case reports, and duplicate articles. Based on the criteria above, two reviewers (YR and SW) scanned the titles and abstracts independently to select potential eligible articles and then reviewed the full texts to decide whether they were consistent with our study. Discrepancies were discussed and resolved through consultation with a third reviewer (JW). We utilized a Preferred Reporting Items for Systematic Reviews and Meta-Analyses (PRISMA) flowchart for detailed study selection. The two reviewers independently extracted data regarding details of type of study, study population, participants, intervention, and outcome measures and duration on the basis of a self-developed data extraction form. Disagreements were resolved through discussion with all reviewers.

### 2.4. Risk of Bias in Individual Studies

We assessed risk of bias of the included studies according to the Cochrane Handbook for Systematic Reviews of Interventions (Chapter 8.5) (Higgins 2011), which contained seven aspects: random sequence generation, allocation concealment, blinding of participants and investigators, blindness of outcome assessments, incomplete outcome data, selective outcome reporting, and other biases. Baduanjin exercise made blinding of participants and investigators impossible. We judged low, unclear, or high bias for each aspect on the basis of the Cochrane criteria.

### 2.5. Data Synthesis and Analysis

We used Review Manager 5.2 (RevMan 5.2) for data analysis. We analyzed the statistics by the means of the weighted mean difference (WMD), with 95% CI. The heterogeneity of included studies was assessed by *Q* and *I*^2^ test statistics. As for* Q* statistics, *P* < 0.05 was considered to have significant difference. We conducted random effects models for meta-analysis when significant heterogeneity existed (*P* < 0.05 and *I*^2^ > 50%) among included studies. Otherwise, fixed effects models were applied. Funnel plots were used for evaluating publication bias when more than 10 studies were identified.

### 2.6. Influence Analysis

Influence analysis was performed by Stata V.13.0 software to assess whether the single study would affect the overall result.

## 3. Results

### 3.1. Description of Studies

**Figure 1:**
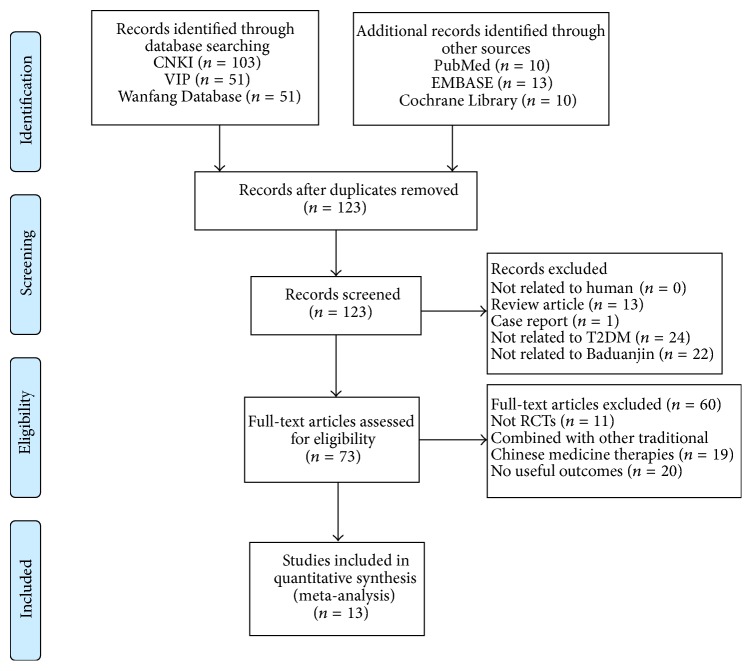
Study selection flow diagram.

We identified 238 potentially relevant articles. After screening titles and abstracts, 115 articles were excluded owing that they were nonclinical studies, expert experience, or case reports. We reviewed the remaining 73 studies, 60 were excluded because they did not meet with our inclusion criteria, 11 of which were not RCTs, 19 articles combined with other traditional Chinese medicine therapies, and 20 articles were excluded because the outcome index did not meet the demand. Therefore, 13 articles [22–34] involving 782 participants met our inclusion criteria. The screening process was summarized in the PRISMA flow diagram (see Figure 1).

### 3.2. Study Characteristics

The 13 studies [22–34] included 782 participants, 393 of whom were in the experimental group and 389 were in the control group, ranging from 37 to 73 years old. All studies were conducted in China. All studies included were two-group parallel designed studies. The duration of studies lasted from 6 weeks to 6 months, and there were 5 studies [24, 26, 28, 33, 34] taking 3 months and 1 study [32] took 6 weeks to research, while 2 articles [27, 29] finished study after 4 months and 5 articles [22, 23, 25, 30, 31] after 6 months. In these included trials, Baduanjin plus conventional therapy was in comparison with conventional therapy alone. Adverse effects were not reported in the included studies. Detailed characteristics of included studies are listed in Table 1.

### 3.3. Risk of Bias

**Figure 2:**
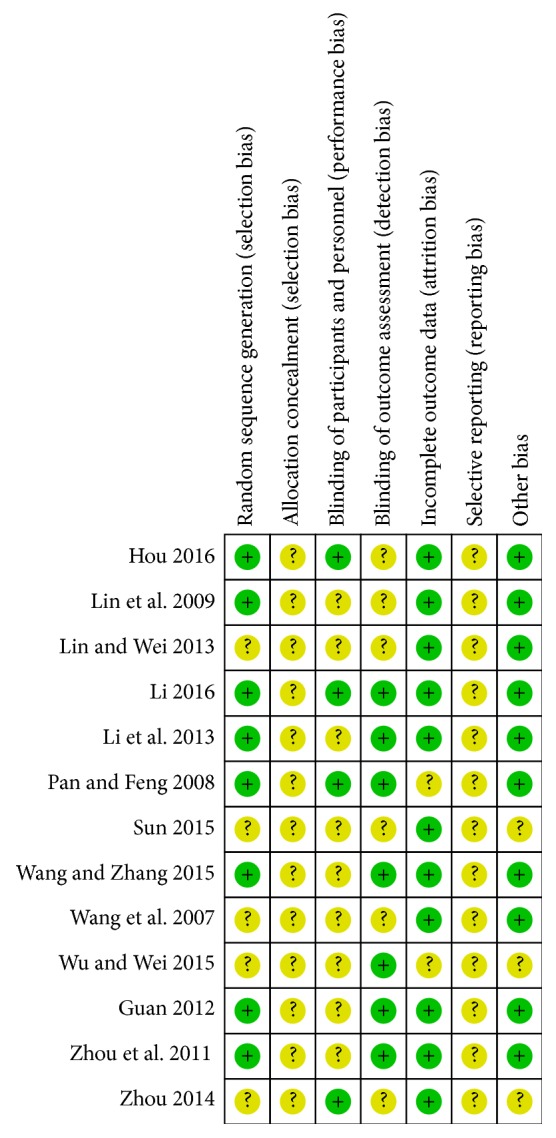
Risk of bias summary of included studies.

We utilized Cochrane Handbook for Systematic Reviews of Interventions (Chapter 8.5) (Higgins 2011) to evaluate risk of bias for each included article. The studies included all claimed randomization, while only 8 trials [22, 23, 27–30, 32, 34] reported concrete methods of random sequences generation. None of studies mentioned allocation concealment that 13 of which were reported to have unclear risk of bias. Four trials [22, 24, 30, 34] illustrated blinding of participants and personnel and 7 trials [22, 23, 28, 29, 32–34] blinded outcome assessment. Two studies [22, 33] did not provide required information and detailed data. Since study protocols were not available, selective reporting was identified as an unclear risk in all included studies (see Figure 2).

### 3.4. Efficacy Assessment

#### 3.4.1. Glycosylated Hemoglobin

**Figure 3:**
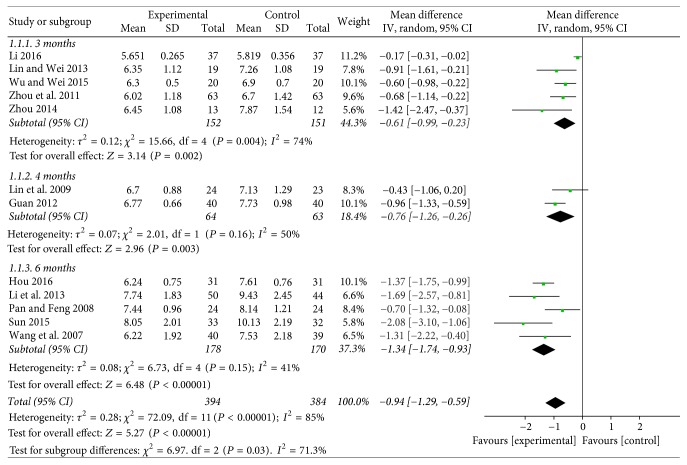
Funnel plot of glycosylated hemoglobin.

Thirteen trials [22–34] with a total of 782 patients reported the level of glycosylated hemoglobin. Five included studies compared the effect on 3 months of Baduanjin intervention plus conventional therapy with routine treatment, which indicated that 3 months of Baduanjin exercise had lower glycosylated hemoglobin compared with patients in control group (WMD = −0.61; 95% CI: −0.99 to −0.23; *P* = 0.002), but not a favorable effect compared with 4 months (WMD = −0.76; 95% CI: −1.26 to −0.26; *P* = 0.003). The combined effect showed that Baduanjin therapy for 6 months (WMD = −1.34; 95% CI: −1.74 to −0.93; *P* < 0.00001) had a significantly better effect on glycosylated hemoglobin than the duration of 3 or 4 months (see Figure 3).

#### 3.4.2. Fasting Blood-Glucose

**Figure 4:**
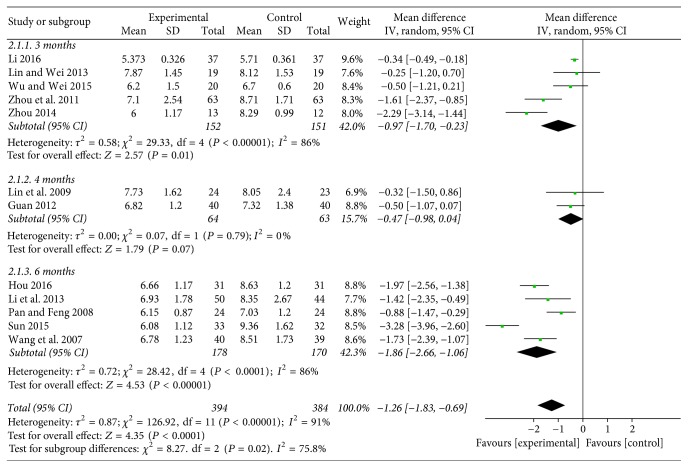
Funnel plot of fasting blood-glucose.

Thirteen studies [22–34] assessed fasting blood-glucose in 782 patients. Three studies compared the effect on Baduanjin intervention of 3 months with the control group; the combined effect showed that 3 months of Baduanjin exercise had a better effect than that of conventional therapy (WMD = −0.97; 95% CI: −1.70 to −0.23; *P* = 0.01). Two studies compared the effect on 4 months of Baduanjin exercise with the conventional control group and the result indicated that Baduanjin therapy for 4 months had a better effect on FPG (WMD = −0.47; 95% CI: −0.98 to 0.04, *P* = 0.07). Five studies compared the effect on FPG of 6 months of Baduanjin intervention with conventional therapy, the combined effect illustrated that Baduanjin therapy for 6 months decreased FPG significantly (WMD = −1.86; 95% CI: −2.66 to −1.06; *P* < 0.00001) (see Figure 4).

#### 3.4.3. Postprandial Plasma Glucose

**Figure 5:**
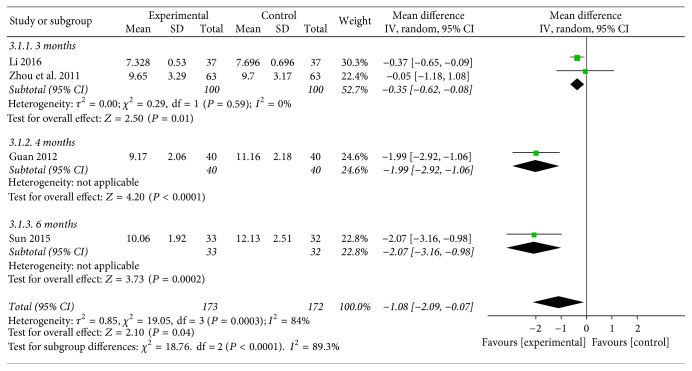
Funnel plot of postprandial plasma glucose.

Pooling the data of five studies [28, 29, 31, 32, 34] that evaluated the postprandial plasma glucose, two studies of Baduanjin intervention for 3 months compared the effect on postprandial plasma glucose with conventional therapy group, which revealed significant effects favoring Baduanjin exercise (WMD = −0.35; 95% CI: −0.62 to −0.08, *P* = 0.01). One study compared the effect of 4 months of Baduanjin plus conventional treatment with conventional therapy alone and the result showed the better effect of Baduanjin plus conventional treatment (WMD = −1.99; 95% CI: −2.92 to −1.06, *P* < 0.00001). One trial compared the effect of Baduanjin therapy plus conventional therapy for 6 months with conventional monotherapy; the statistically significant decrease on postprandial plasma glucose could be found (WMD = −2.07; 95% CI: −3.16 to −0.98, *P* = 0.0002) (see Figure 5).

#### 3.4.4. TC

**Figure 6:**
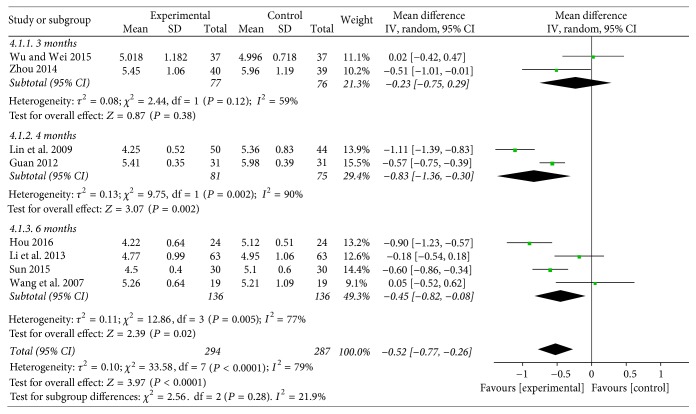
Funnel plot of TC.

Eight studies [22, 23, 25, 26, 28, 30, 32, 34] reported the total cholesterol (TC). Two studies compared the effect on Baduanjin plus conventional intervention for 3 months with conventional therapy alone; the combined effect showed that significant differences were not observed (WMD = −0.23; 95% CI: −0.75 to 0.29, *P* = 0.38). Two studies compared the effect on Baduanjin plus conventional intervention for 4 months with conventional monotherapy; the combined effects of eight trials showed that Baduanjin exercise lowered the TC significantly in patients with diabetes when comparing with conventional control (WMD = −0.83; 95% CI: −1.36 to −0.30, *P* = 0.002). Four trials compared the effect on Baduanjin plus conventional intervention for 6 months with conventional therapy alone; the combined effects showed that Baduanjin plus conventional treatment had a better effect (WMD = −0.45; 95% CI: −0.82 to −0.08, *P* = 0.02) (see Figure 6).

#### 3.4.5. TG

**Figure 7:**
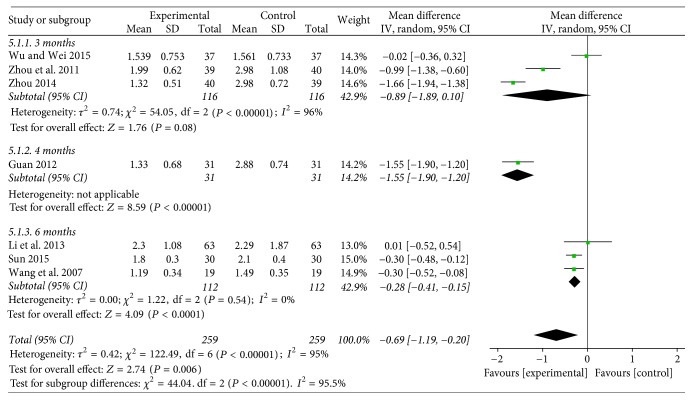
Funnel plot of TG.

Seven studies [25, 26, 28–30, 32, 34] with 518 patients applied TG as an outcome measure. Baduanjin plus conventional therapy for 3 months described a clinical reduction on TG (WMD = −0.89; 95% CI: −1.89 to 0.10, *P* = 0.08). One study of 4 months of Baduanjin plus conventional therapy expressed the same result (WMD = −1.55; 95% CI: −1.90 to 1.20, *P* < 0.00001). The remaining three trials reflected that Baduanjin exercise plus conventional therapy lowered the TG (WMD = −0.28; 95% CI: −0.41 to 0.15, *P* < 0.00001) (see Figure 7).

#### 3.4.6. HDL-C

**Figure 8:**
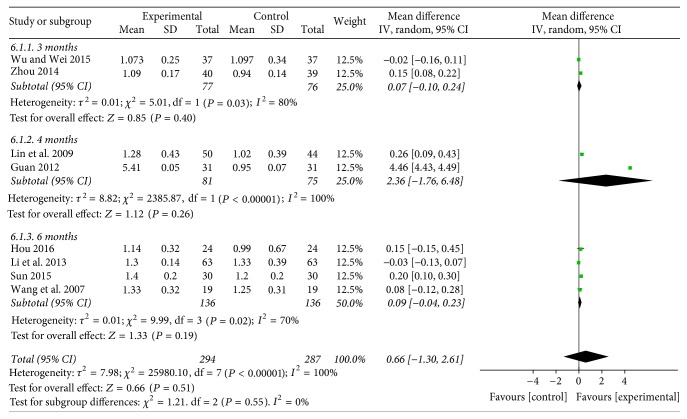
Funnel plot of HDL-C.

Data were extracted from eight studies [22, 23, 25, 26, 28, 30, 32, 34] including 581 patients to assess HDL-C. In two trials, Baduanjin plus conventional therapy for 3 months were in comparison with conventional monotherapy, which revealed that Baduanjin plus conventional therapy for 3 months promoted the level of HDL-C (WMD = 0.07; 95% CI: −0.10 to 0.24, *P* = 0.40). Moreover, the results indicated significant difference favoring Baduanjin plus conventional therapy for 4 months (WMD = 2.36; 95% CI: −1.76 to 6.48, *P* = 0.26), when compared with conventional therapy. Four studies compared the effects of Baduanjin plus conventional therapy with conventional therapy alone, the combined effects showed that the former raised HDL-C (WMD = 0.09; 95% CI: −0.04 to 0.23, *P* = 0.19) (see Figure 8).

#### 3.4.7. LDL-C

**Figure 9:**
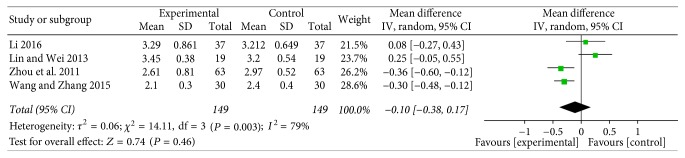
Funnel plot of LDL-C.

LDL-C was reported in four trials [26, 28, 32, 34] of 298 patients, with significant between-study heterogeneity (WMD = −0.18; 95% CI: −0.30 to −0.06, *P* = 0.003). We did not conduct a subgroup analysis on accessing LDL-C, owing that the duration of the four studies was 3 months (see Figure 9).

### 3.5. Publication Bias

**Figure 10:**
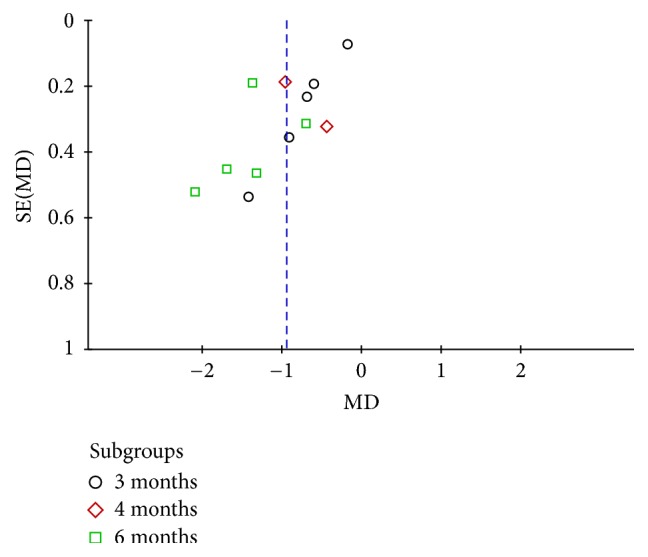
Funnel plot of glycosylated hemoglobin.

**Figure 11:**
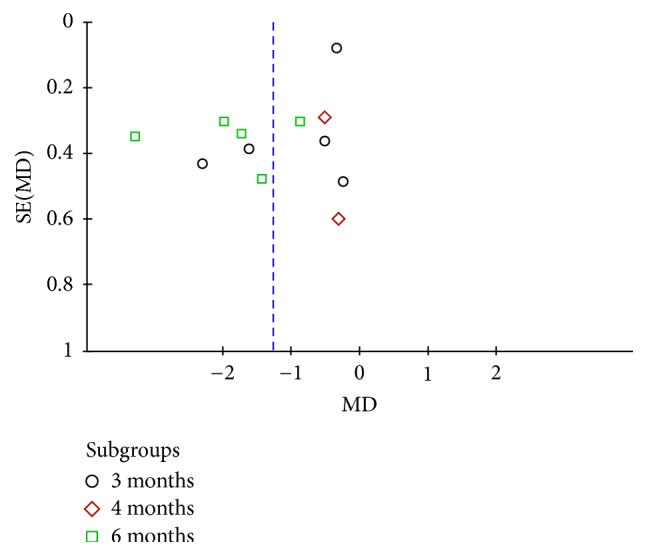
Funnel plot of fasting blood-glucose.

**Figure 12:**
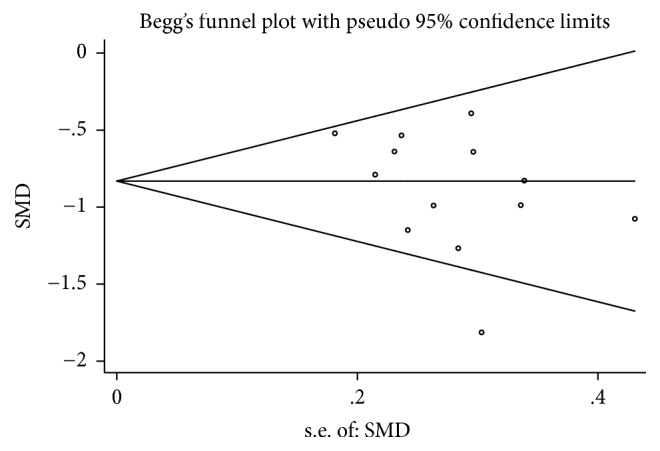
Begg's funnel plot of glycosylated hemoglobin.

**Figure 13:**
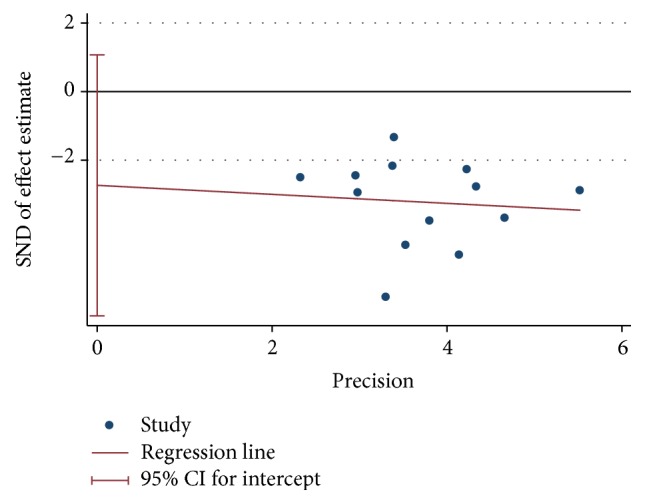
Eegg's funnel plot of glycosylated hemoglobin.

**Figure 14:**
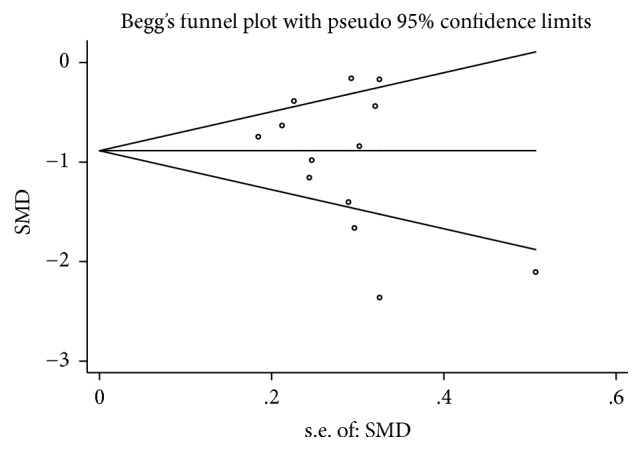
Begg's funnel plot of fasting blood-glucose.

**Figure 15:**
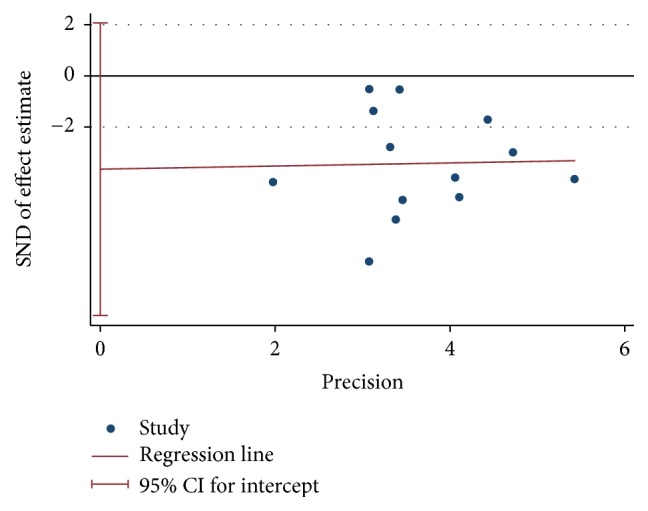
Egger's funnel plot of fasting blood-glucose.

To evaluate publication bias, we conducted the funnel plot for the included studies of glycosylated hemoglobin and fasting blood-glucose (see Figures 10 and 11). Owing to insufficient details of other outcomes, we did not conduct the funnel plot. The asymmetrical figure reflected that potential publication bias might have an influence on results of meta-analysis. And no publication bias was detected by Begg's and Egger's test. (*P* values: 0.142 and 0.188, resp.) (see Figures 12, 13, 14, and 15).

### 3.6. Influence Analysis

**Figure 16:**
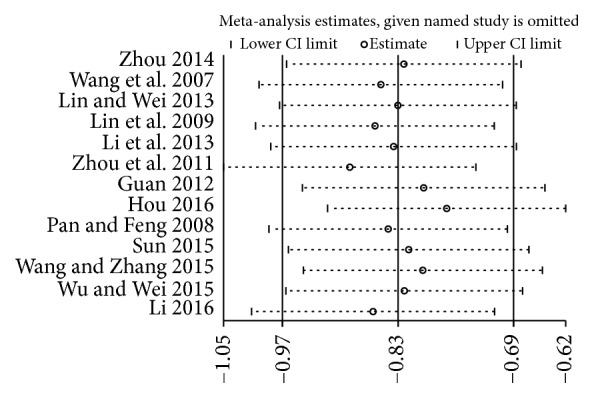
Influence analysis of glycosylated hemoglobin.

**Figure 17:**
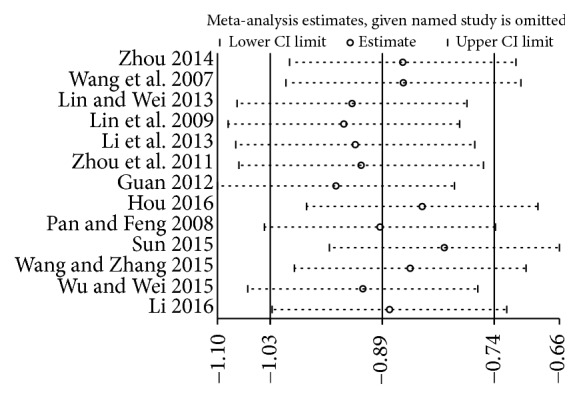
Influence analysis of fasting blood-glucose.

We conducted the influence analysis for the included studies of glycosylated hemoglobin and fasting blood-glucose. No single study affected the overall result by influence analysis (see Figures 16 and 17).

### 3.7. Adverse Events

Table 1 showed that there was no mention of adverse events in all included studies. Besides, the dropout data were not reported.

## 4. Discussion

### 4.1. Summary of Main Results

Baduanjin, one of the most common Chinese Qigong exercises, has existed for more than one thousand years [32]. Considered as a popular and safe community exercise to promote health in China, Baduanjin is easy to grasp and exerts an outstanding effect on strengthening the body [33]. We performed a meta-analysis of data to prove this relevancy: as an auxiliary therapy for diabetic, Baduanjin exercise could lower blood sugar (two hours after meal) and reduce glycated hemoglobin, total cholesterol, triglycerides, and low-density lipoprotein cholesterol levels. And it could raise the level of high-density lipoprotein, which can lower the risk of cardiovascular disease.

### 4.2. Mechanism of Baduanjin

Baduanjin is suitable for the elderly and the weak, since it consists of only eight sections of simple, slow, and relaxing movements [34]. By strengthening various movements of limbs, such as stretching and pitching, and the flow of the internal Qi [34], Baduanjin helps to adjust breathing and achieve the unison of mind and body [35, 36]. When Baduanjin is applied to the treatment of diabetes, it also has obvious changes on some clinical outcome measures closely related to diabetes. Previous study showed that Baduanjin exercise could decrease glucose and HbA1c and improve the immune function in patients with type II diabetes [37]. Further study showed that Baduanjin can effectively regulate and control the level of blood sugar, reduce HbA1c and blood lipids, and improve the level of HDL (high-density lipoprotein) on the basement of conventional treatment [38]. It is reported that after the treatment of Baduanjin, the number of patients with increased VC shows an obvious improvement. The total cholesterol (TC), fasting blood-glucose (FBG), and glycosylated hemoglobin (GHB) levels were decreased under conditions of standing still or being loaded, while high-density lipoprotein cholesterol (HDL-C) level was increased [25]. Furthermore, it is reported that Baduanjin exercises could improve living quality of patients with T2DM through increasing the sensitivity of body to insulin and decreasing the insulin resistance (IR) index of the body [39].

Therefore, Baduanjin exercises can regulate and control the level of blood sugar, reduce HbA1c, blood lipids, and insulin resistance, improve the level of HDL (high-density lipoprotein) and the lipid metabolism in patients with type 2 diabetes mellitus.

### 4.3. The Comparison between the Baduanjin and Other Qigong on Diabetes

When comparing the effect exerted on the patients with type 2 diabetes mellitus, Baduanjin seems better than some other kinds of Qigong. Tai Chi, another kind of traditional Chinese Qigong, failed to show FBG-lowering and HbA1c-reducing effects [40]. Some researchers show that Tai Chi was unable to improve HDL-C [41] and lower TC [42]. Though yoga practice is beneficial in control of blood sugar levels of patients with T2DM [43, 44], this study does not make a preview on the effect that yoga may exert to some worthy measure outcomes such as the level of TC and HDL-C. Though a comprehensive literature search in English and Chinese databases was conducted, no studies identified have compared Baduanjin versus Tai Chi, jogging, or other common exercises. Therefore, whether Baduanjin has more benefits than other exercises was still unclear and this warrants further studies.

### 4.4. Limitations for Research

However, some limitations preclude us from coming to definite conclusions.

Firstly, according to the statement published by the members of the ICMJE in September 2004, all clinical trials are required to be registered before being published [45]. But none of included studies had been registered.

Secondly, the methodological quality of the included RCTs was generally low. (a) Most of them do not describe allocation concealment and blinding, which have a negative effect on the authenticity of the results. (b) The sample size of most of the included studies was relatively small, which often increases the possibility of overestimating intervention benefits. (c) Publication bias may be present.

Thirdly, high clinical heterogeneity could lower the reliability and validity of the research results.

Fourthly, most were published in Chinese journals. It reduced the extrapolation of the results. Simultaneously, many new data have been published. And because of the defect of search strategy, some studies might be left out.

## 5. Conclusion

This study has implications for practice in spite of these limitations, but it is premature to conclude the efficacy of Baduanjin exercise for the treatment of diabetic. Further standardized preparation, rigorously designed RCTs, and large sample size are required.

## Figures and Tables

**Table 1:** Characteristics of included studies.

Included trials	Sample size		Age, mean (SD) (years)		Interventions		Outcomes measured	Adverse events/follow-up
	T	C	T	C	T	C		
Zhou 2014	13	12	Age ranges from 51 to 72	Age ranges from 58 to 80	More than 30-minute Baduanjin exercise for 3 months, with training twice per day	Regular healthcare for 3 months	①, ②	Not mentioned

Wang et al. 2007	40	39	57.8 (7.5)	56.5 (6.9)	60-minute Baduanjin exercise for 6 months; regular drug treatment	Regular drug treatment and healthcare for 6 months	①, ②, ④, ⑤, ⑥	Not mentioned

Lin and Wei 2013	19	19	64.5 (11.5)	60.8 (12.2)	More than 45-minute Baduanjin exercise for 3 months, with training twice per day; regular drug treatment	Regular drug treatment and healthcare for 3 months	①, ②, ④, ⑤, ⑥, ⑦	Not mentioned

Lin et al. 2009	24	23	59.38 (5.29)	55.30 (8.67)	60-minute Baduanjin exercise for 4 months	Regular healthcare for 4 months	①, ②,	Not mentioned

Li et al. 2013	19	19	50..42 (9.68)	52.69 (8.37)	30-minute Baduanjin exercise for the first 3 months and do it consciously for the last 3 months; regular drug treatment	Regular drug treatment and healthcare for 6 months	①, ②, ④, ⑥	Not mentioned

Zhou et al. 2011	63	63	67.4 (9.23)	68.13 (10.64)	30-minute Baduanjin exercise for 3 months	3 to 5 aerobics weekly for 3 months	①, ②, ③, ④, ⑤, ⑥, ⑦	Not mentioned

Guan et al. 2012	40	40	59.20 (8.80)	58.70 (8.30)	60-minute Baduanjin exercise for 4 months; regular drug treatment and healthcare	Regular drug treatment and healthcare for 4 months	①, ②, ③, ⑤	Not mentioned

Hou 2016	31	31	58.82 (6.78)	58.93 (6.47)	Five 30-minute Baduanjin exercise weekly for 6 months; regular drug treatment	Regular drug treatment and healthcare for 6 months	①, ②, ④, ⑤, ⑥	Not mentioned

Pan and Feng 2008	24	24	47 (7)	45 (9)	Five Baduanjin exercise weekly for 24 weeks with training 45 minutes twice per day; regular drug treatment	Regular drug treatment for 24 weeks	①, ②, ④, ⑥	Not mentioned

Sun 2015	33	32	46.1 (11.8)		Two 60-minute Baduanjin exercise and relaxation exercises weekly for 6 months; regular drug treatment	regular drug treatment	①, ②, ③	Not mentioned

Wang and Zhang 2015	30	30	61.7 (6.9)	61.3 (8.4)	Five 20-minute Baduanjin exercise weekly for 6 weeks; regular drug treatment	Regular drug treatment for 6 weeks	①, ②, ③, ④, ⑤, ⑥, ⑦	Not mentioned

Wu and Wei 2015	20	20	63.9 (7.6)	65.3 (6.0)	More than 5 Baduanjin exercise weekly for 3 months, with training three times per day	Regular drug treatment and healthcare for 3 months	①, ②,	Not mentioned

Li 2016	37	37	45.186 (9.360)	44.973 (8.136)	5 Baduanjin exercise weekly for 3 months, with training 30 minutes once or twice per day	Regular healthcare for 3 months	①, ②, ③, ④, ⑤, ⑥, ⑦	Not mentioned

① Fasting plasma glucose; ② glycosylated hemoglobin; ③ 2-hour postprandial blood glucose; ④ total cholesterol; ⑤ triglycerides; ⑥ high density lipoprotein cholesterol; ⑦ low density lipoprotein cholesterol.
